# Supplementary material for: Are carbon emissions trading and green financial instruments synergistic? -Comprehensive quantitative research based on content analysis
Source: PLoS One. 2024 Mar 7;19(3):e0298601. doi: 10.1371/journal.pone.0298601 (PMC10919601; doi:10.1371/journal.pone.0298601)
Supplement: S1 Data — (ZIP) [file pone.0298601.s001.zip › Data/地级市Z_F工作报告.zip!/╦─┤¿/┼╩╓a╗¿╩╨/└┤╫╘╣1⁄2╓┌║┼í╛┬φ┐╦╩2╛▌═°í┐_2015.docx]

各位代表：

　　现在，我代表市人民政府，向大会报告工作，请予审查，并请政协委员和列席同志提出意见。

　　一、2014年工作回顾

　　过去的一年，面对宏观经济持续下行、国际矿价接连冲击、产品市场长期低迷、企业效益整体下滑等严峻形势，在市委的坚强领导下，在市人大、市政协的监督支持下，市政府团结全市各族人民，主动适应新常态，沉着应对破难题，抢抓机遇谋发展，经济实现平稳较快增长，社会保持和谐稳定，民生得到持续改善，主要发展指标高于全国、全省平均水平。全年实现地区生产总值870.85亿元，增长9.3%，增速居全省第7位；全社会固定资产投资616.81亿元，增长13%；社会消费品零售总额245.55亿元，增长12%；公共财政收入62.91亿元，增长7.5%；城镇居民人均可支配收入27322元，增长9.7%，农村居民人均纯收入10960元，增长11.4%；居民消费价格指数上涨2%；城镇登记失业率为3.6%；人口自然增长率为2.7％。；单位生产总值综合能耗预计下降8.4%。

　　（一）全力以赴稳增长，整体实力持续增强稳增长措施落地见效

　　全面落实中央和省上稳增长政策，出台了促进经济增长26条、稳定工业12条、引导房地产业发展15条等措施。增加信贷总量49.8亿元，发行小微企业增信集合债券6亿元，减免税收17.12亿元，提供应急转贷资金17.48亿元，争取用电优惠1.1亿元。新登记民营企业2075家，增长103%；非公有制经济实现增加值417.52亿元，增长10.5%，占全市GDP的47.9%。

　　项目建设成绩突出。8大类33项“急难险重”任务和20项市长重点推进项目进展顺利，110个市级重大项目完成投资202.06亿元，超计划5.3个百分点。白马铁矿选钛、瑞钢渣钢渣铁、东方钛业扩能、枣子坪220KV输变电等项目竣工投运，丽攀高速攀枝花段、龙密路至马家田连接线建成通车，观音岩水电站首台机组并网发电，桐子林水电站三期明渠截流成功；成昆铁路新线攀枝花段、干坝子风电场、金沙水电站、马鞍山水库等项目加快建设；大面山风电场、攀大（理）高速等项目开工建设。

　　财税金融运行平稳。实现全口径公共财政收入116.76亿元，其中税收收入96.18亿元；公共财政支出121.41亿元，增长4.8%。全市银行业本外币各项存款余额803.99亿元、贷款余额661.38亿元，分别比年初增长2.3%、8.1%，存贷比达82.3%，居全省第一位。保险业保费收入18.74亿元，增长12.9%。

　　（二）坚定不移促转型，三次产业协同发展

　　工业经济提质增效。六大工业园区实现规模以上工业总产值864.23亿元，增长14.1%，钒钛高新区建成省级出口工业产品质量安全示范区。四大企业转型发展步伐加快，攀钢本部产值和税金同步上扬，攀煤精煤产量创历史新高，十九冶完成利税4.5亿元，钢城集团经营总收入突破200亿元。攀钢重轨钢产量达140万吨、特种用途无缝钢管填补国内空白，金沙纳米产品跻身军工领域。完成技改投资232.47亿元，增长4.3%，国内最大的脱硝催化剂材料和器件生产基地初步形成，白马球团、顺腾集团创建为省级企业技术中心。13户企业关闭淘汰落后工艺。

　　服务业提档升级。首届中国康养产业发展论坛圆满举行，项目签约240.43亿元，阳光康养产业发展试验区创建规划编制完成。阿署达、普达等项目群加快建设，鑫岛欢乐世界一期建成运营，省级旅游标准化示范城市创建成功，“阳光花城·康养胜地”城市品牌在全国影响扩大，实现旅游总收入150.03亿元，增长46.8%。新启综合物流中心一期和锐华1万吨果蔬气调库冷链物流一期建成，汉风物流入选全省物流10强企业。城市商业网点规划实施，电商发展完成顶层设计，与淘宝大学合作成立的全国首个地级市电子商务学院正式运行。新增限额以上商贸企业110家，新兴服务业增加值占服务业比重超过30%。

　　农业经济稳步发展。成功应对低温雨雪、持续干旱等自然灾害，农林牧渔业总产值50.82亿元，增长4.7%，完成烟叶收购28.8万担。盐边种草养畜、仁和芒果等示范园核心区新品种、新技术集成集约示范任务全面完成，新增万亩现代农业示范片8个、省级农业标准化示范区2个。农产品销路畅通，上线交易网店达716家。新型农业经营主体不断壮大，新增农业产业化省级重点龙头企业3家、省级示范农民专业合作社6家、农民专业合作社150户。农田水利基础进一步夯实，新建高标准农田2.3万亩、高效节水灌溉面积7.2万亩、太阳能提灌站13座。

　　（三）统筹城乡增实力，阳光花城魅力彰显

　　城乡建设切实加强。成功举办全国第三届山地城镇可持续发展专家论坛，城市总体规划获批实施，《中国阳光康养旅游城市发展规划》等专项规划编制完成。城市功能不断提升，花城新区1020亩土地整理初步成型，干坝塘大桥贯通，阳光大道商务办公区段、花城大道电子商务区段铺筑路面，道路骨架基本形成；渡口记忆、福广金城等一批老旧房屋改造项目开工建设。小城镇和新村建设步伐加快，红格、格里坪等8个镇入选全国新型城镇化重点镇名单，平地和白马被列为全省第二批“百镇建设试点”；建成新农村综合体3个，25个幸福美丽新村和50个新村聚居点建设、16个旅游新村改造提升基本完成。城乡路网日益完善，改善通乡通村公路247.9公里，安装公路安全护栏447.18公里，所有行政村提前一年实现通畅目标。

　　城市环境显著改善。智慧城市建设成果丰硕，成功入选“宽带中国”示范城市，成为全国首个全光网城市。沿江景观形象初显，保安营生态功能区治理基本完成，奥林匹克中心、滨水新空间等项目加快建设。“五创联动”深入开展，创建国家森林城市“七大工程”加快推进，第六届全国苏铁学术会议圆满召开；全国文明城市提名资格城市争创工作全面启动；国家园林城市64项创建指标达标37项，城区绿化覆盖率和绿地率稳步提升；省环保模范城市通过复检，二氧化硫排放近10年首次达标，环境空气质量综合监测指数排名全省前列；中国阳光康养旅游城市创建成效突出，成为全省唯一入选中国最佳养老50强城市，名列第24位。

　　（四）锐意创新求突破，发展活力有效激发

　　攀西试验区建设提速。6类8项钒钛资源综合利用项目新进入《西部地区鼓励类产业目录》，19个项目获得2.4亿元省级专项资金支持，钒钛高新区被纳入全省直购电优惠范围。成功推动试验区11个重大科技攻关项目国内外公开招标，我市8个项目进入，获得专项配套资金占全省的79.2%。全国钒钛磁铁矿标准化分技术委员会落户我市，国内首个大型多功能钒钛交易电商平台——攀枝花钒钛交易中心挂牌开市。成功举办首届中国（攀枝花）钒钛产业博览会，吸引800多家企业参会参展，协议交易额418.94亿元，签约投资项目27个274.5亿元。

　　各项改革步伐加快。5个重点领域21项经济体制改革全面实施。攀钢改革力度加大，市国有投资集团、建设集团挂牌成立。全过程绩效预算管理体系建立运行，地税涉税事项在全省率先实现全市通办。PPP试点项目库初步建立。市农商行增资扩股4亿元，市商业银行内江分行挂牌，首批7家社区支行成立。《关于加快培育新型农业经营主体的意见》等4个农村改革文件出台，农村集体建设用地使用权、宅基地使用权确权登记颁（换）证全面完成，米易农村产权交易中心和盐边集体林权流转交易平台建成运营。我市被确定为全省首批两个公立医院改革试点城市之一，4个县级公立医院被纳入全国综合改革试点。米易县成为全省扩权强县试点。工商、税务、质监和公安在政务中心办理事务实现“四证合一”。公车改革启动。教育、文化、人事、办公用房等改革有序推进。

　　开放合作水平提升。围绕钒钛深加工、阳光康养等重点产业精准招商，开展重大投资促进活动15次，招商履约项目270个，到位资金502.9亿元，增长15.7%。钒钛外贸基地信贸合作平台启动，“一次申报、一次查验、一次放行”的全国通关模式加快推进，进出口总额3.01亿美元，增长61%，增速居全省第一位，实际利用外资1.11亿美元。与宝鸡市签订钒钛产业战略合作备忘录，绘制了“钒钛之都”与“钛谷”跨省合作的路线图。

　　科技创新成效凸显。三大国家级创新平台高效运行，钒钛科技孵化器升创为国家级孵化器，成功创建为国家知识产权示范城市。表外矿和低品质矿利用技术取得重大突破，入选品位降低10%，宇航级钒铝合金产业化和钒电池产品开发及应用等项目进入中试研究。获得授权专利943件，万人有效发明专利拥有量居全省第二位，省科技进步奖获奖数量位居全省前列。转化省、市科技成果28件，新增国家高新技术企业13家，高新技术产业产值达160亿元。

　　（五）千方百计惠民生，人民生活不断改善

　　民生保障扎实有力。10大项103个分项民生工程及省、市29件民生实事圆满完成，投入资金达39.75亿元。城镇新增就业17979人，就业困难人员再就业1959人。城乡居民养老保险覆盖面达95%，大病保险全面实施。基本建成保障性住房5971套，改造棚户区5488户、农村危房1599户，安置采空沉陷区居民1028人。投入扶贫资金1.44亿元，“8+8”对口援边、彝家新寨建设试点等扶贫攻坚取得实效，大中型水利水电工程移民搬迁安置有序进行。8.038万农村人饮安全问题得到有效解决，农产品和市民饮食用药安全得到保障。民生满意度指数位居全省第二。

　　社会事业全面发展。新增市级示范幼儿园5所，主城区二十七中小等3所学校建成招生，三区通过国家义务教育均衡发展督导评估，三中、七中创建为全省一级示范性普通高中，攀枝花学院增设康复治疗技术和老年服务与管理专业。省第七届少数民族艺术节圆满举行，省文艺创作培训基地落户我市，市老年大学建成投用，三线建设博物馆即将建成，公共文化服务场所全部免费开放。市中心医院、市三医院分别被确定为全省八大区域医疗中心和六大区域精神卫生中心之一，医患纠纷第三方调处机制建立，法定传染病发病率远低于全省平均水平。单独两孩政策全面实施。成功举办全国桥牌公开赛，市老年体育活动中心开工建设，在第12届省运会上总分跃居第二名。国防动员工作走在全省前列。

　　社会大局保持稳定。社会治理能力综合测评得分全省第一，创建为川滇黔唯一的全国和谐社区建设示范城市。平安攀枝花建设有力推进，基层平安细胞创建深入开展，群众安全感满意度测评位居全省第三。安全生产形势稳中向好，事故起数和死亡人数分别下降31.4%、27.3%。矛盾纠纷调解成功率93.9%，挂牌督办信访积案化解率达100%。全面贯彻民族宗教政策，民族地区和宗教领域和谐稳定。

　　此外，审计、人防、国安、气象、供销、档案、防震减灾、住房公积金管理、地方志、邮政、外事、台务、侨务、妇女、儿童、残疾人、对口援助木里藏区和援建名山地震灾区等各项工作均取得新成绩，电力、通讯、石油等驻攀单位也为全市建设发展作出了新贡献。

　　各位代表，过去的一年，我们面对多重困难，取得了来之不易的成绩！在此，我谨代表市人民政府，向全市各族人民，向驻攀解放军、武警官兵、政法干警和民兵预备役人员，向给予政府工作大力支持的人大代表、政协委员、各民主党派、工商联、无党派人士、人民团体和社会各界人士，向各位老领导、老同志，向来攀创业发展的建设者、投资者，表示衷心的感谢并致以崇高的敬意！

　　我们也清醒地认识到，全市经济社会发展还面临不少困难和问题：地区生产总值、规模以上工业增加值、城镇居民人均可支配收入预期目标虽经努力但仍未实现；部分企业生产经营困难，新引进的重大项目不多，新兴产业对经济增长的支撑力不强，做大总量与加快转型的任务仍然紧迫而艰巨；一些群众生活困难，全面小康依然存在“短板”；社会矛盾较多，不稳定因素仍然存在；政府职能转变还不到位，部分工作人员服务意识不强、履职效率和能力有待提升。对此，我们将勇于担当，统筹施策，切实改进并全力解决。

　　二、2015年主要任务

　　今年，是完成“十二五”规划的收官之年，也是全面深化改革的关键之年和全面推进依法治市的开局之年。世界经济总体复苏的疲软态势难有根本改观，全国、全省经济迎来以增速放缓、方式转变、结构优化、动力转换为主要特征的新常态，我市面临的外部环境在短期内难有明显好转。但同时，中央坚持稳中求进的工作总基调，继续实施积极的财政政策和稳健的货币政策，重点推进“一带一路”、长江经济带、西部开发等发展战略，省委、省政府高度重视攀西试验区建设、南向通道构建和康养产业发展，为我市发展带来多重利好。

　　今年政府工作的总体要求是：以党的十八大和十八届三中、四中全会精神为指导，深入贯彻省委十届四次、五次全会及市委九届八次全会精神，围绕在全省率先全面建成小康社会和建设新攀西，主动适应经济发展新常态，坚持稳中求进总基调，以提高经济发展质量和效益为中心，以转变发展方式为主线，以深化改革、创新驱动为动力，以保障和改善民生为重点，以依法治市、从严治党为保障，全力以赴“稳增长、促转型、抓改革、转作风、防风险、保稳定”，坚定不移推进“三个加快建设”“三个走在全省前列”，坚持不懈打好“六大硬仗”，科学谋划好“十三五”发展规划，努力促进全市经济持续健康发展、社会大局和谐稳定。

　　全市经济社会发展的主要目标为：地区生产总值增长8%、力争9%；全社会固定资产投资增长10%、力争10.5%；社会消费品零售总额增长11%、力争12%；公共财政收入增长5%、力争6%；城镇居民人均可支配收入和农村居民人均纯收入分别增长10%、力争11%；居民消费价格指数增速控制在3.5%以内；城镇登记失业率控制在4%以内；人口自然增长率控制在4％。以内；单位生产总值综合能耗下降2%。

　　围绕上述目标，重点做好以下工作：

　　（一）加快产业转型升级，确保经济稳定增长

　　以新型工业化为主导，做强工业经济。着力产业升级，做大钒钛、机械制造、太阳能、风能、生物等特色产业，做强钢铁、矿业等优势产业，重点发展五大高端成长型产业，确保规模以上工业增加值增长9%、力争10%。强化“一企一策”，帮助大企业加速结构优化、产品升级、品牌培育。加强对中小企业发展的政策、资金、信息等服务，促进非公有制经济健康发展。推进各产业园区错位发展、集聚发展，推动钒钛高新区创建国家高新区。坚持创新驱动，积极创建国家级创新型试点城市，注重发挥企业家才能，支持企业开发新工艺、新技术、新产品，培育国家高新技术企业6家。加快无线城市网络、云计算数据中心等信息系统建设，推进工业化和信息化深度融合。强化煤电油运等要素保障，稳妥推进煤矿复工复产。

　　以康养产业为龙头，做大第三产业。高标准规划、引进和实施一批康养项目，促进信息、文化教育、旅游、绿色、养老等产业融合发展，争创国家级康养产业发展试验区。完善康养、旅游设施和配套服务，推动阿署达、普达等项目取得新进展，办好国际自驾游博览会、欢乐阳光节等活动，确保旅游总收入突破200亿元。加快密地现代商贸物流园、纷美冷链物流产业园等项目建设，力促金江、总发等物流园开工。建设大宗工业品、优特农产品电子商务交易平台，抓好阳光康养旅游电子商务布局和电子商务、快递进农村综合示范建设，确保电子商务园区建成投用，力争攀枝花钒钛交易中心交易量突破100亿元。推进零售、餐饮、住宿等传统服务业提档升级。促进房地产业健康发展。

　　以国家现代农业示范区为载体，做优特色农业。发展壮大现代农业五大主导产业，狠抓优良品种繁育推广，大力推进米易早春蔬菜、盐边种草养畜等现代农业示范园建设，提高早春蔬菜、水产品效益，扩大畜牧业规模，提升林业产业竞争力，做响特色农产品和优质烤烟品牌。加快培育新型农业经营主体，大力推广标准化生产，引导扶持农业龙头企业做优做强，促进农民专业合作组织、家庭农场、专业大户等规范发展。扎实推进梅子箐水库、太阳能提灌、高效节水等基础设施项目，建设高标准农田2万亩。拓展农产品市场，拓宽增收渠道，促进农民持续增收。

　　以重大项目为支撑，发挥投资拉动效应。围绕中央和省上资金投向，抓紧谋划、推进和新建一批带动性强、影响面广的重大项目，发挥投资对经济增长的关键作用。坚持重大项目推进机制，完善“绿色服务通道”，确保桃树坪风电场、万家山光伏发电一期、西宇生物等项目竣工投产，加快推进钛材加工园、攀钢重轨扩能、中国重汽矿用车生产线、白马铁矿采选扩能改造、旭鼎发动机钒钛铸造件、缅气入攀、观音岩引水、苴却砚文化街区等一批重点项目，开工建设丰源矿业、回龙沟220kV变电站等一批重点工程。

　　以开源节流为主线，做好财税金融工作。进一步优化财政收入结构，着力培植新兴骨干税源，加强税收征管，夯实增收基础。严格预算管理，加强专项资金整合，加大对科技、教育、医疗、社会保障等领域的投入。严控政府债务规模和风险，推进PPP项目试点，引导社会资金进入重点建设领域。支持市外金融机构在攀设立分支机构，鼓励现有金融机构在县（区）及乡镇增设分支机构和网点，深化银政企对接，扩大信贷规模，充分发挥金融支持小微企业和实体经济发展的杠杆作用。大力引进股权投资类机构，支持符合条件的企业利用多层次资本市场和债务融资工具开展直接融资。

　　（二）加速新型城镇化进程，提升城市整体品质

　　以推进城乡基本服务一体化为目标，加快基础设施建设。按照“显山露水、依山就势、错落有致、城在花中”的总体思路，以城市总体规划为引领，加快完善各片区控制性详规。以路网骨架完善、基础设施配套和项目引进为重点，强化建设资金、用地等保障，加速花城新区连线成片开发。推进创业路、原市建二公司等老旧房屋改造，加快沿江景观、干坝塘天星湖景观、山地森林公园等重点项目建设，完善城市管网、路灯等公用设施。积极开展第一、二批“百镇建设试点行动”，启动实施第三批省级试点镇建设，推动小城镇加速发展。抓好20个幸福美丽新村、30个新村聚居点建设，加快已建新村的设施配套。

　　以夯实四川南向开放门户基础为目标，加速构建交通枢纽。启动“十三五”干线公路网、国道线位和航运发展等相关规划，争取将丽攀昭遵铁路纳入国家《铁路“十三五”发展规划》并加快前期工作。如期完成S310线格福路改建、鱼塘至机场路连接线等工程，加快推进成昆铁路新线攀枝花段、攀大（理）高速、综合客运中心等建设，力争开建三堆子跨江大桥，抓好攀枝花至盐源、攀枝花至宜宾沿江高速等项目前期工作。完成G108线、S214线等国省干线公路大中修，进一步整治危病桥梁，推进县乡道改善和重要乡镇连接公路建设。

　　以塑造阳光花城品牌为目标，持续推进“五创联动”。狠抓污染治理和总量减排，强化环境监管，完成钢钒公司炼铁厂新3号烧结机烟气脱硫系统改造、9个农村环境连片整治等任务，严格落实水资源保护政策，确保空气质量进一步改善、城市集中式饮用水水源地水质100%达标。抓好森林生态体系、林业产业体系、生态文化体系重点工程和项目建设，深化生态县（区）创建工作。强化城市管理委员会作用，有序推进城市绿化、花化、亮化、美化，分类收集处理城市生活垃圾，初步建成智慧城市公共服务平台，实现城市建设个性化、管理智能化和服务人性化目标。积极争创全国文明城市。

　　（三）加大改革开放力度，充分释放发展活力

　　加快攀西试验区建设，促进资源综合利用。坚持先行先试，进一步突破工矿废弃地复垦利用、直购电、资源配置等政策瓶颈，在项目建设、产业链延伸等方面取得新进展。围绕试验区“1+5”政策体系，细化落实措施，引导战略资源高效开发利用。发挥各创新平台作用，加大钒钛领域重大关键技术攻关力度，推动首批重大科技攻关招标签约项目加快实施，做好第二批项目招标。用好试验区“一资金三基金”，吸引战略投资，促进高新产业、新兴产业加速发展。扶持小微企业发展，促进大众创业。通过股权期权激励、提供创业条件等方式，培养和引进更多创新创业团队及高层次人才。

　　深化体制机制改革，不断释放改革红利。支持攀钢、攀煤、十九冶等大企业改革发展，加快市属国有企业集团重组整合，发展混合所有制经济，健全市场运营管理体系框架。加大投融资体制改革力度，进一步发挥市场对资源的配置作用。深化部门预算管理改革，积极开展政府购买服务试点。加速推进农村土地承包经营权确权登记颁证工作，引导农村土地经营权有序流转。抓好户籍制度改革，促进流动人口基本公共服务均等化。稳步推进公立医院改革，积极探索医养融合，完善基层卫生运行机制。深化人才体制改革，健全人才激励保障机制。探索收入分配制度改革，完成机关事业单位职工养老保险和公车改革等任务。深化科技、教育、文化、统计等各项改革。

　　发挥区位优势，扩大开放合作成效。积极融入泛珠三角经济圈、“一带一路”、长江经济带、东盟自由贸易区及中印孟缅经济走廊，加强与台湾、东南亚、南亚等地区和产业关联城市合作，促进川滇黔十二市州互利发展，培育一批跨区域的产业集群。完成攀枝花铁路口岸迁建任务，筹建钒钛海关保税仓库，促进鞍钢国贸、东方钛业等企业进出口稳步增长，推动攀钢、十九冶等企业开展境外投资、国际工程承包和劳务合作，确保外经外贸取得新突破。

　　增强招商引资实效，确保更多项目落地。抓好第十五届西博会、中国钒钛产业博览会及首届中国康养产业发展论坛等签约项目的对接服务，促成项目全面落地。办好东盟—南亚商品交易暨投资洽谈会，借中外知名企业四川行、川欧经济合作交流暨项目洽谈会等平台，重点围绕攀西试验区、康养产业、现代农业示范区等开展投资促进活动，多点发力，精准招商，引进更多旗舰企业、重大高端项目、配套产业，实现招商引资到位资金530亿元。

　　（四）持之以恒改善民生，切实增进群众福祉

　　推进民生工程，解决群众实际困难。加大资金投入，完善推进机制，办好民生大事，确保完成省、市民生任务。做好农村劳动力转移、培训和城镇困难群体、高校毕业生等就业工作，确保城镇新增就业1.6万人、失业人员再就业5000人、就业困难人员就业1200人。统筹推进城乡社会保障体系建设，不断扩大“五险”覆盖范围。开工建设保障性住房6300套以上，完成城镇危旧房、棚户区和农村危房改造任务，加强新（改）建农村民居抗震设防工作。全面推进精准扶贫，抓好边远民族地区对口帮扶，做好大中型水利水电工程移民安置，帮助困难群众改善生产生活条件。加强食品药品安全工作。

　　发展社会事业，打造区域教育、文化、卫生高地。大力发展学前教育，均衡发展义务教育，提升高中阶段教育水平，抓好3所中职学校国家级改革发展示范学校建设，加强特殊教育和民族教育，支持攀枝花学院特色发展，积极推进教育信息化建设。完善公共文化服务体系，加快实施文化“七大工程”， 深入开展文化活动，积极申报“中国三线建设文化遗产城”。着力建设卫生 “六大中心”，加快花城新区医院、市妇幼保健院（迁建）等基础设施建设，推进人口健康信息化建设，提升城乡医疗卫生服务水平。深入实施全民健身条例，积极申办省级以上体育赛事。大力创建省级质量强市示范城市。加强国防动员、民兵预备役和“双拥”工作。做好审计、人防、国安、出入境检验检疫、气象、档案、地方志、外事、台务、侨务、民族宗教、工商、供销、妇女、儿童、残疾人等工作。

　　创新社会治理，促进社会和谐稳定。以增强群众安全感满意度为目标，坚持系统治理、依法治理、综合治理、源头治理，深化平安创建，坚持严打整治，提升社会治理能力。畅通诉求渠道，健全依法维权和矛盾纠纷化解机制，推动信访积案化解，切实维护群众合法权益。高度重视安全生产，强化现场监管和隐患治理，坚决遏制重特大安全事故发生。

　　三、政府自身建设

　　2014年，市政府严格落实中央八项规定和省、市各项规定，深入开展党的群众路线教育实践活动，为民务实清廉的政府形象进一步树立。共减少政府机构3个，调整优化重要职责6项，新建和修订各类规章制度146个。取消、暂停、下放市级行政审批41项，政务服务标准化试点通过验收。按要求公开市级部门预算、决算和“三公”经费，市政府门户网站在全国301个地级市绩效评估中位列第15名，“攀枝花发布”获评四川十佳政务微信。办结人大代表建议139件、政协提案306件，均无不满意意见。

　　2015年，我们以“打铁还需自身硬”的态度和“咬定青山不放松”的决心，切实加强政府自身建设，以法治、高效、廉洁的政府形象取信于民。

　　全面依法行政。严格按照法定权限和程序行使权力、履行职责，坚决执行市人大及其常委会的决议决定，自觉接受人大工作监督、法律监督和政协民主监督，认真听取各民主党派、工商联、无党派人士和人民团体的意见建议，自觉接受人民群众和新闻舆论的监督，构建办事依法、遇事找法、解决问题用法、化解矛盾靠法的法治良序。加快行政执法体制改革，推进行政综合执法，坚决查纠执法不力、执法扰民、执法寻租等行为。深入推进政务公开，实行重大项目听证、重要事项公示、重点工作通报等制度。加强智库建设和决策咨询工作。

　　坚持高效履政。深化行政审批制度改革，取消一批行政审批权项，统一公布行政许可项目清单并实行动态调整，清理取消非行政许可审批事项。加大企业投资领域简政放权力度，进一步下放项目核准权限。认真践行“现场办、马上办”“你有事、我加班”等承诺，推进并联审批，简化手续、减少环节、优化流程。牢固树立“空谈误国，实干兴邦”理念，紧扣落实链条，深化绩效管理，对重点项目和重大任务定标准、定时限、定责任，立说立行，强力攻坚。加强督促检查，严肃查处“庸懒散浮拖”等行为，严格逗硬奖惩，加大问责力度，确保办成一批大事、实事、难事。

　　始终从严治政。全面履行“一岗双责”，层层落实党风廉政建设责任制，完善惩治和预防腐败体系，从源头上防控廉政风险。严格执行中央八项规定和廉洁自律各项规定，深化“9+4”专项治理和5项集中整治，健全反“四风”长效机制，保持“有案必查、有腐必惩”的高压态势，打造一支严要求、硬执行的公务员队伍。

　　各位代表，时势日新，催人奋进！让我们紧密团结在以习近平同志为总书记的党中央周围，在省委、省政府和中共攀枝花市委的坚强领导下，集聚正能量，适应新常态，动真碰硬，敢于担当，创新实干，为完成全年目标任务和在全省率先全面建成小康社会而努力奋斗！
